# Health Promotion and Disease Prevention in Public Housing Areas: A Scoping Review

**DOI:** 10.3390/ijerph22111624

**Published:** 2025-10-25

**Authors:** Iben Engelbrecht Giese, Signe Lykke Justsen, Vibeke Brinkmann Løite, Stine Hangaard

**Affiliations:** 1 Steno Diabetes Center North Denmark, Aalborg University Hospital, 9260 Gistrup, Denmark; vbl@rn.dk (V.B.L.); svh@hst.aau.dk (S.H.); 2 Department of Health Science and Technology, Aalborg University, 9260 Gistrup, Denmark; slykkejustsen@gmail.com

**Keywords:** health promotion, disease prevention, public housing, scoping review, digital health

## Abstract

Residing in public housing is associated with adverse health outcomes, partly due to higher prevalence of unhealthy lifestyle behaviors linked to lower socioeconomic status. Health promotion and disease prevention interventions can mitigate these disparities but are often underutilized due to accessibility barriers and low health literacy. Delivering interventions directly within public housing areas may enhance reach and effectiveness. However, synthesized knowledge of such interventions remains limited. This scoping review aimed to identify and summarize available evidence on health-promoting and disease-preventive interventions in these settings. The review followed the Preferred Reporting Items for Systematic reviews and Meta-Analyses extension for Scoping Reviews (PRIMA-ScR) guidelines. A systematic search was performed in PubMed, CINAHL, Embase, and Scopus. Articles were screened using predefined criteria. Intervention details, key findings, and digital components were extracted and categorized. 31 articles were included, covering eight intervention categories: (1) Health promoter programs, (2) Nutrition programs, (3) Health screenings, (4) Health promotion messages, (5) Physical activity programs, (6) Mental health programs, (7) Oral health programs, and (8) Other health interventions. Five articles incorporated digital components. This review highlights the value of resident involvement, demonstrated by positive outcomes in interventions with strong community engagement. Despite promising effects, digital health components were underutilized, representing a missed opportunity for scalable, cost-effective interventions.

## 1. Introduction

Residing in a public housing area is associated with adverse health outcomes [1]. The concept of public housing varies internationally and encompasses different governance models. In many contexts, public housing is not only provided directly by government authorities but also by non-profit housing organizations that operate to deliver affordable housing to low-income populations [2,3,4]. Non-profit-administered housing is often characterized by long-term tenancy, relatively stable resident populations, and a focus on community-oriented management [2,4]. Given these features, and to ensure a more consistent contextual basis, the present review defined public housing as affordable housing accessible to all individuals, primarily administered by non-profit organizations [3]. Public housing is primarily inhabited by individuals with lower socioeconomic status (SES), who often experience significantly adverse health outcomes compared to those with higher SES [5]. This health disparity is evidenced by an increased prevalence of chronic diseases, such as cardiovascular disease and type 2 diabetes, adverse mental health, obesity, and elevated mortality rates [6,7,8]. These disparities can be explained by the fact that individuals with a lower SES often exhibit a higher prevalence of unhealthy lifestyle behaviors, including smoking, physical inactivity, and poor dietary patterns [1,9,10,11,12,13,14]. Hence, addressing these health disparities requires targeted interventions regarding lifestyle behavior that consider the challenges faced by public housing residents.

Health promotion and disease prevention constitute essential intervention strategies for addressing these issues. Such interventions can reduce the risk of developing new health conditions, prevent the worsening of existing chronic diseases, and enhance individuals’ self-efficacy in managing their own health [15,16,17,18,19,20]. However, the utilization of such promotive and preventive healthcare interventions by residents in public housing areas is often hindered by multiple accessibility barriers. These barriers include significant geographical distances to healthcare services, which are typically located in health centers or similar facilities far from public housing areas [21,22]. In addition, the surrounding environment may lack walkability and perceived safety, making it difficult or unsafe for residents to engage in physical activity or to travel to these services on foot [21,22,23]. Limited public transportation options and time-consuming travel further restrict access [21,22]. Moreover, residents in public housing areas frequently experience limited health literacy, which may affect their understanding of both the importance and the content of health promotion and disease prevention initiatives [24,25]. This limited health literacy can lead to a lack of awareness and comprehension of available healthcare services and how to utilize them effectively [21,24,26]. Consequently, bringing health promotion and disease prevention initiatives closer to public housing areas may enhance accessibility and safety, and ensure that the interventions are more effective and widely utilized.

Despite the importance of health promotion and disease prevention interventions in public housing areas, there is a notable gap in the literature regarding the specific strategies that have been implemented in these settings. To the best of the authors’ knowledge, no literature review has been conducted to gather and synthesize the experiences from such interventions. Therefore, this scoping review seeks to identify and map the available evidence on health promotion and disease prevention interventions in public housing areas. It aims to address the research question: What health-promoting and disease-preventive interventions are identified in public housing areas? Additionally, digital components applied in the interventions will be identified and mapped, as digitalization has had a major impact in health promotion and preventive interventions, demonstrating great potential for increasing health outcomes and ensuring proximity of the interventions [27,28,29,30,31]. However, factors such as poverty, limited digital access and low literacy in public housing areas may affect the feasibility of these components, which makes it important not only to identify them but also to assess their effect [32].

## 2. Materials and Methods

The current scoping review was conducted in accordance with the Preferred Reporting Items for Systematic Reviews and Meta-Analyses extension for Scoping Reviews (PRISMA-ScR) guidelines [33] (Supplementary Material S1). This scoping review was conducted without a preregistered protocol.

### 2.1. Inclusion and Exclusion Criteria

Original peer-reviewed articles that described completed health-promoting and disease-preventive interventions in public housing areas were included. Public housing was understood as non-profit administered, affordable housing, and studies conducted in temporary or exclusively government-owned housing models were excluded. The results of the interventions needed to be presented in the articles to be included. Full-text articles published between 1 January 2004 and 18 September 2024 were considered for inclusion. Articles written in English or any of the Scandinavian languages were accepted. Articles describing interventions in public housing areas exclusively government owned or reserved for specific social groups were excluded to ensure homogeneous contexts. Additionally, articles describing interventions focusing on environment or interior in relation to health promotion were excluded as the review focused solely on interventions targeting the individual. If an article reported on multiple interventions, it was included only if the results for environmental and individual-based interventions were presented separately.

### 2.2. Search Strategy

Initially, a non-systematic search was performed in PubMed, Embase, and Google Scholar to identify relevant search terms, keywords, and index terms to incorporate in the systematic search. Following this, the systematic literature search was performed in four relevant databases: PubMed, CINAHL, Embase, and Scopus on the 18th of September 2024. The search strategy involved a block search comprising two main blocks, using the overall search terms “Public Housing” and “Health-promoting or preventive interventions,” respectively. To broaden the search, we incorporated synonyms for the primary search term, utilizing a combination of MeSH terms and relevant keywords. An experienced research librarian was consulted to validate the search strategy. One review author (SLJ) performed the searches in the databases. For full-electronic search strategy, see Supplementary Material S2.

### 2.3. The Screening Process

First, all articles identified through the systematic search were collected and uploaded into RefWorks 2024, where duplicates were removed. Second, all remaining articles were screened by title and abstract by one review author (SLJ) to assess their eligibility based on the inclusion criteria. In case of uncertainty regarding the relevance of an article, another review author (IEG) was consulted. Third, the remaining articles were assessed for full-text screening (SLJ, IEG). During the full-text screening, reasons for exclusion of articles were registered and presented in a PRISMA flow diagram. Any disagreement between the review authors during the screening process was resolved through discussion or by involving a third author (SH).

### 2.4. Data Extraction and Synthesis

All articles included following full-text screening were read profoundly by one review author (IEG). The table function in Microsoft Word 2024 was utilized to extract data from each included article, following a predefined table format that was agreed upon by three of the review authors (SLJ, IEG, SH). The following article details were extracted: Author, year, population, type of housing, aim of intervention, description of the intervention, digital component (if any), and key findings. In case of uncertainty regarding article details, another review author (SLJ) was consulted. Any disagreement was resolved through discussion or by involving a third reviewer (SH).

Extracted details from the articles were utilized to synthesize categories based on the type of health-promoting or disease-preventive intervention by review author IEG. An article could be included under multiple categories if the intervention contained various aspects relevant to different categories. Separate tables based on the identified categories were created, and the content of each category was summarized. One review author, SH, validated the synthesis of categories. Furthermore, a separate table was created to summarize the articles in which a digital component was identified. This table provided descriptions of the digital elements involved in the interventions.

### 2.5. Ethics Approval

As the review used publicly available data from previously published studies, ethical approval was not required.

## 3. Results

### 3.1. Screening and Search Outcomes

**Figure 1:**
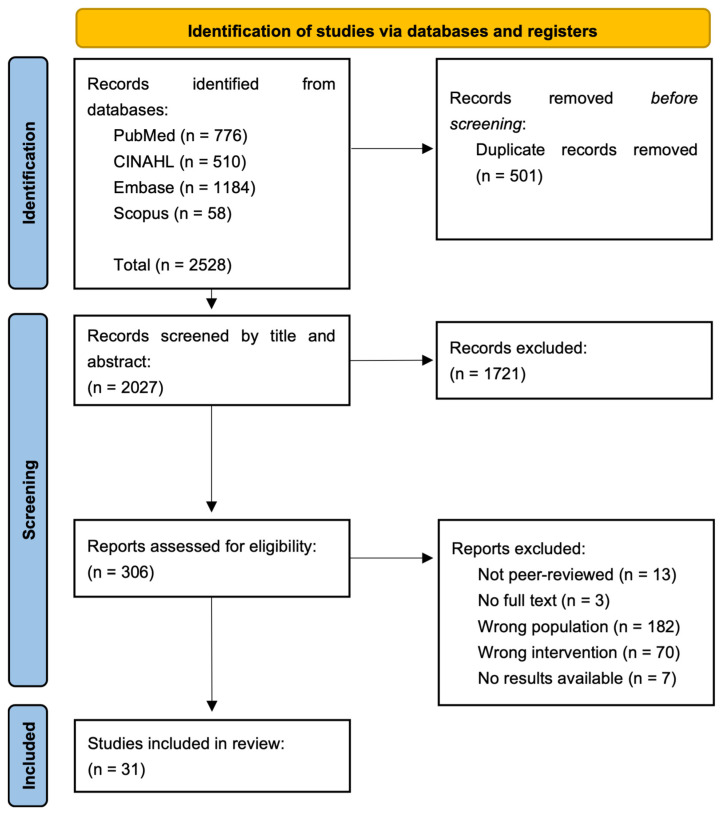
PRISMA flowchart.

A total of 2528 articles were identified in the systematic search, of which 501 were duplicates. After removing the duplicates, the remaining 2027 articles underwent screening on title and abstract, where 1721 articles were excluded due to irrelevance. Subsequently, 306 articles underwent full-text screening, where 275 articles were excluded, mainly because they focused on a wrong population (n = 182) or did not contain a health promotion or disease-preventive intervention (n = 70). After full-text screening, 31 articles were included in the current review. Figure 1 illustrates the full screening process.

### 3.2. Study Characteristics

The characteristics of the included articles are presented in Supplementary Material S3. The included articles were published between 2004 and 2024, of which the majority (n = 25) were carried out in USA (80.6%), two in Denmark (6.5%), two in Canada (6.5%), one in China (3.2%), and one in Australia (3.2%). Five articles (16.1%) included a digital intervention component. A mixed-methods approach was applied in eight (26%) of the included articles. Seven articles (23%) employed a pre-post-test design, and seven (23%) used a qualitative design. A cluster randomized trial design was applied in four articles (13%), while three (10%) articles used a feasibility study design. The remaining two articles employed a non-randomized pilot study design (3%) and a randomized controlled trial design (3%), respectively.

### 3.3. Synthesized Findings

The interventions described in the included 31 articles revealed eight different categories including: (1) Health promoter programs, (2) Nutrition programs, (3) Health screenings, (4) Health promotion messages, (5) Physical activity programs, (6) Mental health programs, (7) Oral health programs, and (8) Other health interventions. Eight articles were included in more than one category.

#### 3.3.1. Health Promoter Programs

Health promoter programs (Table 1) included training residents to become community health advocates. In many cases, residents applied to participate in the health promoter programs [34,35,36,37,38,39,40,41,42], and they often reflected the broader resident population by having the same linguistic and cultural background [36,37,38,40]. The training content commonly included, leadership skills, needs assessments, health promotion activities (e.g., exercise, nutrition, mental health, health screenings, smoking cessation, and navigating healthcare services), self-management theories, and chronic disease education such as diabetes, hypertension, and asthma [34,35,36,37,38,39,40,41,42]. Following training, four studies reported that health advocates conducted needs assessments among residents and implemented health promotion activities based on identified priorities [34,35,37,41]. The health advocates were generally responsible for organizing activities, recruiting participants, and facilitating implementation [34,35,36,37,38,39,40,41,42]. The duration of training ranged from 30 to 56 h, and the subsequent community-based activities lasted from four weeks to two years. Several studies reported that the involvement of community health advocates contributed to increased community engagement, enhanced understanding of health promotion and disease prevention, improved trust, and better access to health services [34,35,36,37,39,41]. Community activities were often successfully implemented due to the engagement and leadership of the community health advocates [34,35,36,37,41]. Only one study reported challenges, primarily related to insufficient training and unclear role definitions for the health advocates [42]. Five of the nine studies reported positive health-related changes, such as reduction in blood glucose, blood pressure and weight, improvements in oral hygiene, and increases mental health [36,38,39,40,41].

#### 3.3.2. Nutrition Programs

Nutrition programs (Table 2) included nutrition education, food-related skill development, interactive gardening, hands-on gardening and cooking, cooking demonstrations, easy access to fruits and vegetables, participatory theater show about making healthy choices, motivational interviewing counseling, cookbooks with healthy recipes, health promotion messages, weight management strategies, and strategies for eating healthy on a budget [35,43,44,45,46,47,48,49,50,51,52]. The frequency of the interventions varied from once a week to twice a week and lasted up to one year. One study included a “booster” session at the end of the program [49]. Several studies reported an increase in the average servings of fruits and vegetables [35,44,45,48], while only one study found no change [49]. Three studies reported an overall decrease in average total caloric intake, consumption of sugar-sweetened beverages and foods, and frequency of fast-food consumption [35,47,49]. Across many studies, participants experienced increased knowledge of gardening and healthy eating and willingness to try fruits and vegetables [44,46,47,52]. One study reported that the program improved social connections and reduced exposure to gangs and drugs [50].

#### 3.3.3. Health Screenings

Health screenings (Table 3) contained life-style questionnaires, screening for psychosocial service needs, and physical examinations measuring, for example, blood pressure, glycated hemoglobin A1c, weight, height, waist circumference, and fitness level [35,40,43,53,54,55]. The screenings were offered to identify health risks such as diabetes [35,40,43,53,54,55]. The screening results were often shared with the participants through a dialogue with the health screener [35,40,43,53,55]. Those at risk were advised to consult a general practitioner or referred to relevant health programs [35,53,55]. Health screenings were primarily held at community centers [35,40,43,53,54] and offered approximately once or twice a week for a maximum of one year. In two studies, residents perceived the health screenings as valuable [53,55], whereas one study noted a lack of meaningful support and follow-up from healthcare professionals [55]. Several studies found decreases in BMI, healthier life-style behaviors, and improvements in mental well-being [35,40,43,53]. One study reported that high attendance at the screenings was linked to active community leaders [54].

#### 3.3.4. Health Promotion Messages

Health promotion messages (Table 4) focused on HPV vaccination, cervical cancer screening, and sugar-sweetened beverages and foods [51,56]. The messages had an educational purpose targeting self-efficacy and motivation, and included photos, web-links, advice, and factual information [51,56]. One study delivered the messages through Twitter [56], while the other used SMS and other social media platforms [51]. The messages were delivered once a day or every other day for one month. Most participants perceived the health promotion messages as a suitable educational strategy [51,56], but no significant impact on the participants’ health behaviors or intentions were reported [56]. One study reported that messages targeting motivation were rated lowest in terms of satisfaction [51].

#### 3.3.5. Physical Activity Programs

Physical activity programs (Table 5) included walking groups, women-only exercise activities, gentle exercise programs, workshop sessions focusing on exercise and its benefits, and physical activities delivered through fun and entertaining formats [35,47,48,50,52,53,57,58]. One program used prizes and acknowledgements to motivate the participants [57]. The frequency of the interventions varied from five times a week to one session per month. Some studies reported high participant satisfaction with the physical activities and workshop content [47,50,52,53]. Programs that included walking groups were associated with significant increases in minutes walked per day and enhanced social connections among neighbors [35,57]. Additionally, two studies focusing on adolescent girls found increases in physical activity levels [48,59]. One study reported that women-only exercise had high attendance, and participants appreciated the opportunity to join activities where they felt safe, which provided personal time and social opportunities [58].

#### 3.3.6. Mental Health Programs

Mental health programs (Table 6) were delivered through group sessions or open workshops that focused on specific themes such as aging, depression, social isolation, men’s health, social support, and health topics like diabetes [53,60]. The frequency of sessions ranged from weekly to once every six to eight weeks. Participants in these studies generally perceived the programs as effective, reporting improved social interactions, and increased mental well-being [53,60].

#### 3.3.7. Oral Health Programs

Oral health programs (Table 7) included motivational interviewing, which were used to analyze the participants situation and develop plans for oral hygiene, combined with practical training in tooth brushing and flossing techniques [61,62]. Moreover, one program also included health fairs with practical oral hygiene activities, question sessions, and campaign messages on oral hygiene to reinforce the motivational interviewing [61]. The interventions lasted six months. Participants in both studies demonstrated significant improvements in oral hygiene (plaque scores, Gingival index, and brushing/flossing skills) and improvements in Oral Health-related Quality of Life [61,62].

#### 3.3.8. Other Health Interventions

The category other health interventions (Table 8) included a smoking cessation program [40], a sexual health program [63], and a social-enhancing activity [64].

The smoking cessation program included a weekly supply of nicotine replacement therapy and personal behavioral counseling [40]. It was held in the local community center. The intervention lasted four weeks. About 30.7% of the participants achieved cessation, but no significant change in exhaled carbon monoxide levels among the participants was reported [40].

The sexual health program included educational workshops supplemented with written material focusing on sexually transmitted infections, safe sex behaviors and symptoms, combined with few practical demonstrations and role-playing scenarios [63]. It was held at the community centres and lasted 30 to 45 min each time. Participants were satisfied with the sexual health program and expressed and demonstrated new skills and increased confidence [63].

Social-enhancing activities such as bus trips, guided tours, and lunch were organized for residents [64]. These activities were held two times over a four-month period. Participants expressed enhanced social interaction, which fostered a sense of community [64]. However, linguistic and cultural differences posed a significant challenge [64].

#### 3.3.9. Digital Components

Five of the included articles utilized digital components as part of their interventions (Table 9). Velez et al. and Allen et al. provided health promotion information and advice via SMS or social media, while Schwinn et al. delivered an internet-based health promotion program [48,51,56]. Deville-Stoetzel et al. used automatically generated reminder messages to inform residents about health promotion workshops, whereas Ahluwalia et al. conducted motivational interviews with included residents over the phone and used educational videos about fruits and vegetables [44,54]. None of articles reported results directly related to the effect of using a digital component.

## 4. Discussion

The current scoping review aimed to identify and map the available evidence regarding health-promoting and disease-preventive interventions in public housing areas. The interventions were categorized into eight themes, with the most covered theme being nutrition education (36%), followed by health promoter program (30%) and physical activities (26%).

Nine of the included articles involved training residents to become health promoters in their own public housing area [34,35,36,37,38,39,40,41,42]. This involvement of residents in developing and implementing health promotion and disease-preventive interventions could be crucial for their success. A review by Cyril et al. explored the level of community engagement and health outcomes in health promotion interventions in urban areas. They found that a high level of community engagement was associated with positive health outcomes compared to interventions with low community engagement [65]. This finding is supported by several reviews, which found health promoters to be effective in health promotion in different settings [66,67,68]. The findings suggest that empowering residents to take an active role in health promotion within their communities can lead to more effective and sustainable interventions. Additionally, training residents as health promoters could be a cost-effective way to extend the reach of health interventions, particularly in resource-limited settings [69].

Only five (16%) of the included studies incorporated a digital component, and none of them reported outcomes directly related to the digital element. Although studies in other settings have shown promising effects on health outcomes related to health promotion and disease prevention when digital components are included [27,28,29,30,31], their use within public housing remains limited and insufficiently evaluated, indicating a need for greater scientific focus within this area. Digital health interventions may offer flexibility and potential to tailor content to residents’ needs and circumstances [70], which could be beneficial in public housing areas, where health needs and access to resources vary [5,21]. However, their implementation may be challenged by factors such as limited access to devices and low digital literacy [21,26]. Therefore, applicability of digital components in this context should be approached with caution, and future research is needed to determine under which conditions, and for whom, digital interventions can be both feasible and effective.

A significant number of studies within this scoping review reported the effects of their interventions through narrative or qualitative syntheses rather than quantifiable measures. While this may limit the ability to directly compare outcomes across studies [71], narrative syntheses and qualitative approaches are valuable within health promotion research because they can capture nuance and contextual complexity that quantitative measures may overlook [72,73]. However, the diversity in reporting formats and methodological approaches poses challenges for consistency, reproducibility, and generalizability of findings [71]. This suggests that the current evidence base may not yet be fully mature and highlights the need for methodological approaches that better integrate both qualitative and quantitative data to enable more comprehensive and comparable assessments of intervention effects.

The current scoping review has several limitations. One significant limitation is the variability in the definition of public housing across different countries. Public housing, also referred to as social housing, is subject to diverse interpretations and implementations depending on national policies, economic conditions, and cultural contexts. To minimize variability, public housing was defined as affordable housing accessible to all individuals and not exclusively government-owned. However, as this is a scoping review, the emphasis on homogeneity may have inadvertently constrained the breadth of the evidence base, thereby challenging the exploratory nature of the scoping methodology [74]. Thus, our findings may not be fully transferable to settings with different governance structures, such as entirely government-owned housing (e.g., the USA, England, and Canada) [75,76]. Future research should compare interventions across different public housing models to capture the full diversity of contexts in which health promotion interventions are implemented. Another limitation is that this review focused only on individually oriented interventions. This has excluded environmental or policy-level strategies, which are known to be highly effective in health promotion [77,78]. Future research should therefore examine multiple levels of intervention to capture the full scope of health promotion efforts in public housing contexts.

## 5. Conclusions

The current scoping review identified a range of health-promoting and disease prevention interventions in public housing areas, with nutrition education, health promoter programs, and physical activity being the most common. Notably, resident involvement through training as health promoters emerged as a key strategy, supporting evidence that community engagement enhances intervention effectiveness and sustainability. These findings highlight the importance of empowering residents to take active roles in shaping health initiatives within their communities. Despite the growing relevance of digital health, digital components were rarely incorporated and insufficiently evaluated, indicating a need for cautious consideration and further research to determine their feasibility in contexts characterized by limited access and digital literacy. Qualitative and narrative approaches offer valuable contextual insight, but the diversity of methods and reporting formats limits consistency and comparability across studies. Future research should examine different models of public housing and apply multi-level strategies—combining individual, environmental, policy-level, and potentially digital approaches—to strengthen the evidence base and support scalable health promotion interventions.

## Figures and Tables

**Table 1:** Overview of the articles included in the category “Health promoter programs”. The authors, year of publication, content of the intervention, training duration, and key findings are stated.

Author (Year)	Intervention	Training Duration	Key Findings
Bowen et al. (2015) [34]	Resident health advocates were recruited among the residents and trained in basic information about health conditions, leadership, community organization and local health sources. They developed and implemented workshops and collected health surveys.	14 weeks of 4 h sessions.	The resident health advocates provided valuable health information and resources to their communities, improved residents’ access to health services, and built trust within the community.
Bowen et al. (2018) [35]	Health living advocates were recruited among the residents and trained in research processes, weight management and patient privacy. They assisted residents in health screenings, promoted community activities and led walking groups.	14 weeks	The residents considered the activities useful and achievable, and the social contact within the activity was perceived as particularly helpful.
Brown et al. (2011) [36]	Healthy family advocates were recruited among the residents and trained in healthcare and social services, empowerment, disease-specific knowledge and communication skills. They provided personal support, navigating healthcare services and providing educational materials on various health topics.	30 h	The healthy family advocates reported being able to build trust with residents, which facilitated better health outcomes. Healthy family advocates reported enhanced access to healthcare services, improved health behaviors, and greater health knowledge among the residents.
Freeman et al. (2020) [37]	Community health workers recruited among the residents and trained in chronic disease management, motivational interviewing, mental health first aid, and smoking cessation. They assessed the individual residents’ goals, motivation, created action plans, supported, provided health education on chronic disease, and assisted in healthcare services.	35 h	They reported significant reductions in the percentage of participants who needed but could not access health services, a place to exercise, job training or employment programs, and education services.
Hassaballa et al. (2015) [38]	Diabetes health ambassadors recruited among the residents and trained in diabetes self-management education, support, diabetes care services, and community organization. They engaged residents in activities, provided education and linked them to community resources.	30 h	The intervention resulted in significant reductions in HbA1c levels, weight, and diastolic blood pressure.
Jassal et al. (2020) [40]	Two resident leaders were recruited and underwent tobacco-cessation training program. They recruited and held a 4-week cessation program including nicotine replacement therapy, screening for psychosocial services, and making referrals to relevant health programs.	Two weeks	The resident leaders demonstrated a score of 100% knowledge post-tobacco cessation training. 30.7% of the residents achieved cessation based on exhaled carbon monoxide.
Lai et al. (2017) [39]	Resident leaders were recruited and trained to become health promoters. They implemented health-promoting activities to engage residents.	4 h	The program led to increases in physical and mental health and improved neighbor cohesions among residents.
Oliver et al. (2024) [42]	Concierges, often residents, received training in COVID-19 safety, de-escalation, and emotional intelligence. They were placed in the foyers, provided residents with up-to-date information about COVID-19, health restrictions, and health services.	n/a	Engagement with Concierges varied. The program faced challenges leading to variable service delivery. Concierges expressed a need for more comprehensive training and clearer role definitions.
Wolff et al. (2004) [41]	Community health advocates selected among residents were trained in leadership skills, community resources, conflict resolution, healthcare, cancer and alcohol and drug abuse. They conducted a needs assessment among the residents and implemented activities.	7 sessions over 7 weeks	The program enhanced residents’ understanding of health promotion and disease prevention, and increased community engagement.

**Table 2:** Overview of the articles included in the category “Nutrition programs”. The authors, year of publication, content of the intervention, frequency, duration, and key findings are stated.

Author (Year)	Intervention	Frequency	Duration	Key Findings
Agarwal et al. (2019) [43]	Referrals to community resources, such as dietitians and cooking classes, following health assessments, with the usual access to health services through a family physician.	Weekly	n/a	The intervention led to significant improvements in blood pressure, positive changes in lifestyle risk factors, and a reduction in BMI.
Ahluwalia et al. (2007) [44]	Motivational interviewing counseling focusing on fruit and vegetable intake, cooking book, dietary education materials and videos on fruit and vegetable consumption.	5 times	8 weeks	The program resulted in a significant increase in fruit and vegetable intake. Higher participation and more recipes tried were associated with greater increase in fruit and vegetable intake. Participants reported high satisfaction with the intervention.
Bowen (2018) [35]	Easy access to fruit and vegetables through a Fresh Truck and cooking demonstrations.	Weekly and every three months.	One year	The intervention produced a significant decrease in BMI, an increase in mean fruit and vegetable intake, and a reduction in fast-food intake.
Cotter et al. (2018) [45]	Nutrition education, including mindful eating and eating healthfully on a budget.	Weekly	4 weeks	Participants increased their weekly vegetable consumption, and 85% reported moderate or significant improvements in their health.
Grier et al. (2015) [46]	Interactive gardening or nutrition education followed by hands-on gardening for youth.	Weekly	10 weeks	Participants expressed positive impressions, increased confidence in gardening skills, and greater willingness to try fruits and vegetables.
Kuross & Folta et al. (2010) [47]	Participatory theater shows about making healthy choices, cooking games, and science experiments.	3–6 times	10 weeks	63% reported learning useful new information regarding making healthy lifestyle choices, such as incorporating more fruits and vegetables into their diets.
Schwinn et al. (2014) [48]	Web-based health promotion sessions, completed by mother-daughter dyads, focusing on topics like making healthy decisions and the benefits of family meals.	3 times	3 weeks	The program led to increased vegetable intake.
Shankar et al. (2007) [49]	Combination of nutrition education and food-related skill development.	Twice a week	3 weeks	No significant change in average fruit and vegetable servings was observed, but total calorie intake decreased.
Strunin et al. (2013) [50]	Basketball sessions along with educational workshops covering topics such as nutrition targeting young boys	Weekly	n/a	The program brought the boys together and helped them avoiding gangs and drugs.
Velez et al. (2023) [51]	Messages focusing on reducing sugar-sweetened beverages and foods through social media or SMS.	Every other day	1 month	82.9% found the messages useful. Messages on reducing dietary sugars rated the lowest acceptability.
Whittemore et al. (2014) [52]	Interactive education sessions focusing on topics like nutrition aiming at preventing type 2 diabetes.	Every other week to monthly	6 months	The education was well-received by the residents. They engaged actively in discussions, shared personal experiences, understood the content and applied it to their daily lives.

**Table 3:** Overview of the articles included in the category “Health screenings”. The authors, year of publication, content of the intervention, who conducted it, and key findings are stated.

Author (Year)	Intervention	Conducted by	Key Findings
Agarwal et al. (2019) [43]	Comparison of weekly drop-in health assessments (e.g., blood pressure, diabetes risk, and fall risk) with usual health services access through family physician.	Community paramedics.	The intervention led to significant improvements in blood pressure, positive changes in lifestyle risk factors, and a reduction in BMI.
Aselton et al. (2011) [53]	Regular health screenings, such as blood pressure monitoring.	Nursing students.	Participants reported high satisfaction with the personalized health screenings, which contributed to improved health.
Bowen et al. (2018) [35]	Monthly health screenings for blood pressure, smoking and diabetes risk and received referrals to relevant healthcare services.	n/a	The intervention resulted in a significant decrease in BMI and positive changes in lifestyle risk factors.
Deville-Stoetzel et al. (2021) [54]	Monthly health assessment including blood pressure and cardiovascular risk evaluation.	Trained volunteers.	High attendance to health assessment was linked to active community leaders.
Jassal et al. (2020) [40]	Screening for psychosocial healthcare needs once during a 4-week period.	Resident leaders and research staff.	The most frequently reported needs were employment, substance usage treatment, and mental health. More psychosocial services were desired by 80%.
Møller & Merrild (2020) [55]	One health check, including a physical examination, such as blood pressure, glycated hemoglobin A1c, and waist circumference, and questionnaires regarding lifestyle choices.	Health professionals.	Most participants reported the health checks as valuable, although they noted a lack of support and follow-up.

**Table 4:** Overview of the articles included in the category “Health promotion messages”. The authors, year of publication, content of the intervention, frequency, duration and key findings are stated.

Author (Year)	Intervention	Frequency	Duration	Key Findings
Allen et al. (2020) [56]	Women receiving messages on Twitter with an educational content focusing on HPV vaccination and cervical cancer screening.	Daily	1 month	There were no significant changes in HPV knowledge or vaccination intentions, but the intervention was considered an acceptable method of delivering health messages.
Velez et al. (2023) [51]	Delivering of messages focusing on reducing sugar-sweetened beverages and foods through social media or SMS.	Every other day	1 month	82.9% found the messages useful, but motivation-focused messages received the lowest score. SMS was the most accepted way of delivery.

**Table 5:** Overview of the articles included in the category “Physical activity program”. The authors, year of publication, content of the intervention, frequency, duration, and key findings are stated.

Author (Year)	Intervention	Frequency	Duration	Key Findings
Aselton (2011) [53]	Twice a year, new nursing students conducted needs assessments and implemented exercise activities such as gentle exercises, relaxation therapy, and seated jazz dancing.	Once a week	5 years	Participants reported high satisfaction with the various activities, which contributed to improved health and well-being.
Bowen et al. (2018) [35]	Walking groups.	Weekly	1 year	The intervention led to a significant decrease in BMI and an increase in minutes of walking per day.
Krieger et al. (2009) [57]	Walking groups, stretching exercises, and walking information champaign.	5 times a week	n/a	The proportion of participants meeting moderate weekly activity levels increased from 61.5% to 80.8%. Social connectedness was significantly enhanced. Participants also reported fewer days of suboptimal physical and mental health.
Kuross & Folta et al. (2010) [47]	Participatory theater shows about making healthy choices, music and dance, and sports.	3–6 times	10 weeks	63% reported learning useful new information regarding making healthy lifestyle choices, such as increasing physical activity.
Marinescu et al. (2013) [58]	Women-only exercise activities and women-only swimming.	n/a	18 months	Participants appreciated having women-only activities where they felt comfortable and safe while exercising, and the program provided personal time and social opportunities.
Schwinn et al. (2014) [48]	Web-based health promotion sessions, completed by mother-daughter dyads, focusing on topics like making healthy decisions.	3 times	3 weeks	The intervention led to increased physical activity.
Strunin et al. (2010) [59]	Adolescent girls participating in two different types of sessions: a physical activity session and a health education session including themes such as body image and goal setting.	2 times a week	3 years and 4 months	The girls increased their confidence and became more physically active.
Whittemore et al. (2014) [52]	Interactive education sessions, focusing on topics like exercise, aiming at preventing type 2 diabetes and overcoming exercise barriers.	Every other week to monthly	6 months	The education was well-received by the residents. They engaged actively in discussions, shared personal experiences, understood the content and applied it to their daily lives.

**Table 6:** Overview of the articles included in the category “Mental health programs”. The authors, year of publication, content of the intervention, duration and key findings are stated.

Author (Year)	Intervention	Duration	Key Findings
Aselton et al. (2011) [53]	Twice a year new nursing students conducted needs assessments and implemented activities such as education in depression and meditation sessions.	5 years	Participants reported high satisfaction with the various activities, which contributed to improved health and well-being.
Gerson et al. (2004) [60]	Group sessions, each focusing on a specific theme, such as depression, physical changes in aging, and social isolation.	49 weeks	Participants considered the groups effective and experienced improvements in social interactions.

**Table 7:** Overview of the articles included in the category “Oral health programs”. The authors, year of publication, content of the intervention, duration and key findings are stated.

Author (Year)	Intervention	Duration	Key Findings
Reisine et al. (2016) [61]	Adapted motivational interviewing combined with practical training in oral hygiene techniques and two health fairs focusing on oral hygiene.	6 months	The program led to significant improvements in oral hygiene.
Reisine et al. (2021) [62]	Comparison of an individual-based intervention, which included counselling sessions and practical training in oral hygiene techniques, to community-based campaigns.	6 months	There was no significant difference in oral health-related quality of life between the individual-based and community-based intervention groups, but oral hygiene and oral health-related quality of life increased in both groups.

**Table 8:** Overview of the articles included in the category “Other health interventions”. The authors, year of publication, content of the intervention, duration, and key findings are stated.

Author (Year)	Intervention	Duration	Key Findings
Jassal et al. (2020) [40]	A tobacco cessation program including nicotine replacement therapy, screening for psychosocial services, and making referrals to relevant health programs.	4 weeks	Only 30.7% achieved cessation.
Gedin & Resnick (2014) [63]	Workshop regarding sexual health including education in sexually transmitted infections and safe sexual behavior.	One time	The participants’ confidence in sexual behavior and knowledge increased.
Srivarathan et al. (2020) [64]	Bus trips, guided tours, lunch and afternoon tea to enhance social interactions.	Two times over a 4-month period.	Participants reported positive social inter-actions, forming new relationships, strengthening existing social ties, and fostering a sense of community.

**Table 9:** Overview of the articles utilizing a digital component as a part of their intervention. The authors, year of publication, and description of the digital component is stated.

Author (Year)	Digital Component
Ahluwalia et al. (2007) [44]	Motivational interviewing over the phone and education video regarding fruit and vegetables.
Allen et al. (2020) [56]	Health-promoting messages using Twitter.
Deville-Stoetzel et al. (2021) [54]	Reminder messages regarding the organization of health promotion workshops.
Schwinn et al. (2014) [48]	Web-based health-promoting program.
Velez et al. (2023) [51]	Health promotion messages using SMS and social media.

## Data Availability

The original contributions presented in this study are included in the article/supplementary material. Further inquiries can be directed to the corresponding author.

## Supplementary Materials

The following supporting information can be downloaded at: https://www.mdpi.com/article/10.3390/ijerph22111624/s1, Supplementary Material S1: PRISMA-ScR Checklist; Supplementary Material S2: Overview of the full database searches; Supplementary Material S3: Characteristics of included articles.

## Abbreviations

The following abbreviations are used in this manuscript:

BMI	Body Mass Index
SMS	Short Message Service
HPV	Humant Papillomavirus
