# Supplementary material for: Health Promotion and Disease Prevention in Public Housing Areas: A Scoping Review
Source: Int J Environ Res Public Health. 2025 Oct 25;22(11):1624. doi: 10.3390/ijerph22111624 (PMC12652447; doi:10.3390/ijerph22111624)
Supplement: Supplementary file 1 [file ijerph-22-01624-s001.zip › Supplementary Material S2.pdf]

Supplementary Material S2. Overview of the full databases search.

Search number	Search details	Results
**PubMed**		
4	("Housing"[MeSH Terms] OR "Public Housing"[MeSH Terms] OR "afford* hous*"[Title/Abstract] OR "communit* hous*"[Title/Abstract] OR "hous* afford*"[Title/Abstract] OR "hous* association*"[Title/Abstract] OR "public* fund* hous*"[Title/Abstract] OR "social* hous*"[Title/Abstract]) AND ("Health Education"[MeSH Terms] OR "health* campaign*"[Title/Abstract] OR "health* educat*"[Title/Abstract] OR "health* intervent*"[Title/Abstract] OR "health* initiativ*"[Title/Abstract] OR "health* prevent*"[Title/Abstract] OR "health* promotion*"[Title/Abstract] OR "health* people program*"[Title/Abstract] OR "life style intervent*"[Title/Abstract] OR "lifestyle intervent*"[Title/Abstract]) AND 2004/01/01:3000/12/31[Date - Publication] AND ("english"[Language] OR "danish"[Language] OR "norwegian"[Language] OR "swedish"[Language])	776
3	("Housing"[MeSH Terms] OR "Public Housing"[MeSH Terms] OR "afford* hous*"[Title/Abstract] OR "communit* hous*"[Title/Abstract] OR "hous* afford*"[Title/Abstract] OR "hous* association*"[Title/Abstract] OR "public* fund* hous*"[Title/Abstract] OR "social* hous*"[Title/Abstract]) AND ("Health Education"[MeSH Terms] OR "health* campaign*"[Title/Abstract] OR "health* educat*"[Title/Abstract] OR "health* intervent*"[Title/Abstract] OR "health* initiativ*"[Title/Abstract] OR "health* prevent*"[Title/Abstract] OR "health* promotion*"[Title/Abstract] OR "health* people program*"[Title/Abstract] OR "life style intervent*"[Title/Abstract] OR "lifestyle intervent*"[Title/Abstract])	1,084
2	"Health Education"[MeSH Terms] OR "health* campaign*"[Title/Abstract] OR "health* educat*"[Title/Abstract] OR "health* intervent*"[Title/Abstract] OR "health* initiativ*"[Title/Abstract] OR "health* prevent*"[Title/Abstract] OR "health* promotion*"[Title/Abstract] OR "health* people program*"[Title/Abstract] OR "life style intervent*"[Title/Abstract] OR "lifestyle intervent*"[Title/Abstract]	366,024
1	"Housing"[MeSH Terms] OR "Public Housing"[MeSH Terms] OR "afford* hous*"[Title/Abstract] OR "communit* hous*"[Title/Abstract] OR "hous* afford*"[Title/Abstract] OR "hous* association*"[Title/Abstract] OR "public* fund* hous*"[Title/Abstract] OR "social* hous*"[Title/Abstract]	39,083
**CINAHL**		
4	S3 AND Publication Date: 20050101-20241231; Language: Danish, English, Norwegian, Swedish	510
3	(MH "Housing") OR (MH "Public Housing") OR “Afford* hous*” OR “Communit* hous*” OR “Hous* afford*” OR “Hous* association*” OR “Social* hous*” AND (MH “Health education+”) OR “Health* campaign*” OR “Health* educat*” OR “Health* intervent*” OR “Health* initiativ*” OR “Health* prevent*” OR “Health* promotion*” OR “Life style intervent*” OR “Lifestyle intervent*”	569

2	(MH “Health education+”) OR “Health* campaign*” OR “Health* educat*” OR “Health* intervent*” OR “Health* initiativ*” OR “Health* prevent*” OR “Health* promotion*” OR “Life style intervent*” OR “Lifestyle intervent*”	262,949
1	(MH "Housing") OR (MH "Public Housing") OR “Afford* hous*” OR “Communit* hous*” OR “Hous* afford*” OR “Hous* association*” OR “Social* hous*”	11,576
**Embase**		
4	'Housing'/exp OR 'Housing affordability'/exp OR 'Afford* hous*':ti,ab,kw OR 'Communit* hous*':ti,ab,kw OR 'Non profit hous*':ti,ab,kw OR 'Nonprofit* hous*':ti,ab,kw OR 'Non commercial hous*':ti,ab,kw OR 'Noncommercial hous*':ti,ab,kw OR 'Hous* afford*':ti,ab,kw OR 'Hous* association*':ti,ab,kw OR 'Public* Fund* Hous*':ti,ab,kw OR 'Social* hous*':ti,ab,kw AND 'Health education'/exp OR 'Health* campaign* ':ti,ab,kw OR 'Health* educat*':ti,ab,kw OR 'Health* intervent*':ti,ab,kw OR 'Health* initiativ*':ti,ab,kw OR 'Health* prevent*':ti,ab,kw OR 'Health* promotion*':ti,ab,kw OR 'Health* people program*':ti,ab,kw OR 'Life style intervent*':ti,ab,kw OR ' ifestyle intervent*':ti,ab,kw NOT 'conference abstract'/it AND [20014-2024]/py AND (english:la OR danish:la OR norwegian:la OR swedish:la)	1,184
3	'Housing'/exp OR 'Housing affordability'/exp OR 'Afford* hous*':ti,ab,kw OR 'Communit* hous*':ti,ab,kw OR 'Non profit hous*':ti,ab,kw OR 'Nonprofit* hous*':ti,ab,kw OR 'Non commercial hous*':ti,ab,kw OR 'Noncommercial hous*':ti,ab,kw OR 'Hous* afford*':ti,ab,kw OR 'Hous* association*':ti,ab,kw OR 'Public* Fund* Hous*':ti,ab,kw OR 'Social* hous*':ti,ab,kw AND 'Health education'/exp OR 'Health* campaign* ':ti,ab,kw OR 'Health* educat*':ti,ab,kw OR 'Health* intervent*':ti,ab,kw OR 'Health* initiativ*':ti,ab,kw OR 'Health* prevent*':ti,ab,kw OR 'Health* promotion*':ti,ab,kw OR 'Health* people program*':ti,ab,kw OR 'Life style intervent*':ti,ab,kw OR ' ifestyle intervent*':ti,ab,kw	1,827
2	'Health education'/exp OR 'Health* campaign* ':ti,ab,kw OR 'Health* educat*':ti,ab,kw OR 'Health* intervent*':ti,ab,kw OR 'Health* initiativ*':ti,ab,kw OR 'Health* prevent*':ti,ab,kw OR 'Health* promotion*':ti,ab,kw OR 'Health* people program*':ti,ab,kw OR 'Life style intervent*':ti,ab,kw OR ' ifestyle intervent*':ti,ab,kw	500,758
1	'Housing'/exp OR 'Housing affordability'/exp OR 'Afford* hous*':ti,ab,kw OR 'Communit* hous*':ti,ab,kw OR 'Non profit hous*':ti,ab,kw OR 'Nonprofit* hous*':ti,ab,kw OR 'Non commercial hous*':ti,ab,kw OR 'Noncommercial hous*':ti,ab,kw OR 'Hous* afford*':ti,ab,kw OR 'Hous* association*':ti,ab,kw OR 'Public* Fund* Hous*':ti,ab,kw OR 'Social* hous*':ti,ab,kw	36,088
**Scopus**		
4	((TITLE-ABS-KEY ("Afford* hous*")) OR (TITLE-ABS-KEY ("Communit* hous*")) OR (TITLE-ABS-KEY ("Nonprofit* hous*")) OR (TITLE-ABS-KEY ("Hous* afford*"))) AND ((TITLE-ABS-KEY ("Health* campaign*")) OR (TITLE-ABS-KEY ("Health* prevent*")) OR (TITLE-ABS-KEY ("Health* promotion*")) OR (TITLE-ABS-KEY ("Life style intervent*")) OR (TITLE-	58

	ABS-KEY ("Lifestyle intervent*"))) AND (LIMIT-TO (LANGUAGE , "English"))	
3	((TITLE-ABS-KEY ("Afford* hous*")) OR (TITLE-ABS-KEY ("Communit* hous*")) OR (TITLE-ABS-KEY ("Nonprofit* hous*")) OR (TITLE-ABS-KEY ("Hous* afford*"))) AND ((TITLE-ABS-KEY ("Health* campaign*")) OR (TITLE-ABS-KEY ("Health* prevent*")) OR (TITLE-ABS-KEY ("Health* promotion*")) OR (TITLE-ABS-KEY ("Life style intervent*")) OR (TITLE-ABS-KEY ("Lifestyle intervent*")))	60
2	(TITLE-ABS-KEY ("Health* campaign*")) OR (TITLE-ABS-KEY ("Health* prevent*")) OR (TITLE-ABS-KEY ("Health* promotion*")) OR (TITLE-ABS-KEY ("Life style intervent*")) OR (TITLE-ABS-KEY ("Lifestyle intervent*"))	185,927
1	(TITLE-ABS-KEY ("Afford* hous*") OR TITLE-ABS-KEY ("Communit* hous*") OR TITLE-ABS-KEY ("Nonprofit* hous*") OR TITLE-ABS-KEY ("Hous* afford*"))	234,171
