# Supplementary material for: Health Promotion and Disease Prevention in Public Housing Areas: A Scoping Review
Source: Int J Environ Res Public Health. 2025 Oct 25;22(11):1624. doi: 10.3390/ijerph22111624 (PMC12652447; doi:10.3390/ijerph22111624)
Supplement: Supplementary file 1 [file ijerph-22-01624-s001.zip › Supplementary Material S3.pdf]

Supplementary Material S3: Characteristics of the included articles.

Author (year)	Country	Study design	Population	Type of housing	Aim	Intervention	Outcome	Results	Digital element
Strunin et al. (2010)	USA	Pre-post test	Adolescent girls aged 9 to 13 years living in public housing.	Public housing.	To promote a lifetime of physical activity and positive health behaviors among adolescent girls.	The program was conducted from July 2002 to October 2005 and consisted of two weekly meetings, each lasting two hours, held after school.<br><br>Each week, the girls participated in two different types of sessions: a physical activity session and a health education session. The physical activity sessions focused on engaging the girls in regular physical activity through fun and entertaining activities, often held outdoors. The goal was to make physical activity a positive and enjoyable experience to	Measuring level of physical activity and behavior together.	Participants increased their health knowledge, self-confidence, and decision-making skills, and became more physically active. However, participation rates were low due to various barriers such as safety concerns, interpersonal conflicts, and lack of community support.	No

Author (year)	Country	Study design	Population	Type of housing	Aim	Intervention	Outcome	Results	Digital element
						motivate the girls to participate.<br><br>The health education sessions aimed to improve the girls' knowledge of risk behaviors and increase their self-confidence, resilience, and self-esteem. Topics included body image, goal setting, self-esteem, development, nutrition, and healthy eating. These sessions included practical activities and group work to actively involve the girls and make the education more relevant and engaging.<br><br>The girls were encouraged to provide input for			

Author (year)	Country	Study design	Population	Type of housing	Aim	Intervention	Outcome	Results	Digital element
						both the health education and physical activity sessions at the beginning of the program, and many of their suggestions were incorporated into the curriculum.			
Wolff et al. (2004)	USA	Qualitative study	Residents in public housing	Public housing.	To provide a description of the development of the community health advocate (CHA) Program, which aimed to enhance health and quality of life.	Selected residents with minimum of six months residency, commitment to positive change, respect from other residents, trustworthiness, and enjoyment working with people were trained to be resident advocates. They were trained in leadership skills, healthcare, conflict resolution, cancer, alcohol and drug abuse,	Improvement in health and quality of life of the residents.	Enhanced understanding of health promotion and disease prevention, greater community involvement and engagement, a heightened sense of safety and security within the building, and an increased sense of pride in their facility. Additionally, the advocates observe improved interpersonal confidence and skills in	No

Author (year)	Country	Study design	Population	Type of housing	Aim	Intervention	Outcome	Results	Digital element
						and community resources.<br><br>Through a needs assessment the resident advocates found three areas of relevance and implemented the following initiatives; Monitor building activities, promote safety and report incidents. Providing group prayer and song three times in a week. Preparing residents for a high school equivalency examination. Annual health fair with health presentations. Monthly coffee club, weekly discussion group and monthly potluck.		interacting with others.	

Author (year)	Country	Study design	Population	Type of housing	Aim	Intervention	Outcome	Results	Digital element
Gerson et al. (2004)	USA	Qualitative study	African American older adults, ≥ 65 years old, cognitively intact, lived independently in public housing.	Public housing.	Development of an enrichment program called "Aging Gracefully" to promote social support, coping with aging, and enhancing positive self-care and health-care behavior.	7 group sessions held every 6 to 8 weeks. Each session focused on a particular theme, such as depression, physical changes of aging, diabetes, isolation around holidays, and a specific approach was used in each theme. The participants were recruited through flyers in their mailboxes and posts on the bulletin boards.	Improvement in social interaction as observed by the group leader. Level of responsiveness and participation in the group sessions.	Improvements in social interaction, a high level of participation and engagement in the group sessions. The participants benefitted from the opportunities to socialize, and several of the participants stated that the groups were effective.	No
Shankar et al. (2006)	USA	Prospective pre-post test.	African-American women of child-rearing age (20 - 50 years). Their parents and grandparents needed to been born in the USA.	Public housing.	To increase fruit and vegetable consumption among African-American women living in public housing.	The nutrition program contained a combination of nutrition education and food-related skill development. The program consisted of six 90-min sessions conducted twice a	Primary outcomes included servings of fruit and vegetables, total calories and percent calories from fat.	Participants' average servings of fruit and vegetable did not change significantly, but of a positive trend associated with class attendance, with high attenders having an average increase of	No

Author (year)	Country	Study design	Population	Type of housing	Aim	Intervention	Outcome	Results	Digital element
						week for 3 weeks, followed by on 90-min "booster" session held 6 weeks later.		0.26 servings, partial attenders increasing by an average of 0.17 servings and non-attenders having a negative change of -0.13 servings. Average total calories decreased among all participants by almost 300 calories. Percent calories from fat decreased by almost 3% and continued to show this decrease.	
Strunin et al. (2013)	USA	Feasibility study	Boys aged 14-17 living in public housing.	Public housing.	To engage adolescent boys in activities that reduce cancer-related behaviors and increase protective behaviors.	The ETM basketball league features weekly games and educational workshops covering topics like violence prevention, substance use recovery, leadership development, and	Substance use, challenges and lessons learned.	The coaches expressed that keeping the boys active help them avoid gangs and drugs. The program had another positive outcome with bringing the boys together across housing development, and	No

Author (year)	Country	Study design	Population	Type of housing	Aim	Intervention	Outcome	Results	Digital element
						nutrition. The teams were established within housing developments, with games and workshops conducted at a nearby Boys and Girls Club.		thereby reducing tension between groups.	
Hassaballa et al. (2015)	USA	Pre-post test	African American women with type 2 diabetes, who live in public housing.	Public housing.	To improve diabetes-related clinical health outcomes for African-American women living in public housing, through the implementation of the Diabetes Care Coordination Program (DCCP).	The Diabetes Care Coordination Program (DCCP) was implemented to provide comprehensive diabetes care to African American women living in public housing neighborhoods. The program utilized the patient-centered medical home (PCMH) model and involved Diabetes Health Ambassadors (DHAs) to deliver services. The DHAs were recruited from the	HbA1c levels, weight, systolic and diastolic blood pressure, body mass index (BMI), LDL cholesterol, and total cholesterol.	There were statistically significant reductions in HbA1c levels, weight, and diastolic blood pressure. HbA1c levels decreased from an average of 7.76% to 7.48%, weight reduced from 199.9 pounds to 197 pounds, and diastolic blood pressure dropped from 82.9 mm Hg to 80.7 mm Hg. Other clinical health outcomes, such as BMI, systolic blood pressure,	No

Author (year)	Country	Study design	Population	Type of housing	Aim	Intervention	Outcome	Results	Digital element
						public housings and had 30 hours of training from a certified diabetes educator. Key components in the DCCP included diabetes self-management education, support for managing diabetes and distress, enhancing access and linkage to care, improving quality of care, community organization and mobilization, and health system transformation. The program offered group education sessions, bi-weekly support groups, and individual consultations with healthcare providers.		LDL cholesterol, and total cholesterol, showed smaller changes.	

Author (year)	Country	Study design	Population	Type of housing	Aim	Intervention	Outcome	Results	Digital element
						Ambassadors played a crucial role in engaging participants, providing education, and linking them to community resources. The DCCP ran for one and a half year.			
Bowen et al. (2015)	USA	Mixed-methods	Residents living in public housing.	Public housing	To engage residents of public housing in improving their health through the Resident Health Advocate (RHA) program and present the results of ten years of recruitment and training within the program.	Each year, a group of public housing residents are trained and certified as RHAs to serve as health resources for their communities. They are then hired as paid interns by the Housing Authority for six to eight months.<br><br>The RHA consist of four phases; Phase 1 included the recruitment, which contained an application round where	The RHAs satisfaction with trainers and the information provided. Weekly activity logs to document community engagement and health promotion activities. Additionally, employment status and the long-term impacts of the program for current and former RHAs. Self-efficacy, knowledge gained, and skills confidence before	Residents trained in the RHA program came from diverse backgrounds with the majority of them being women, between 20 to 40 years old and speaking a primary language other than English.<br><br>The majority of trainees completed the program with a completion rate over 78 % each year. Fewer completed the	No

Author (year)	Country	Study design	Population	Type of housing	Aim	Intervention	Outcome	Results	Digital element
						applicants should explain factors, such as their motivation, past education and volunteer experience. The application process is to identify those residents, who have a strong interest in health and community outreach.<br><br>Phase 2 included RHA training, which consisted of 14 weeks of four-hour sessions. They learnt basis information about health conditions what were especially pronounced in the developments. Furthermore, their received training covering topics such as leadership	and after the training from the RHAs, and furthermore the impact of the program at the community.	internship, but it was often due to taking another job.<br><br>Several former RHAs have gone to a degree program or have gotten a job within the health care sector.<br>RHAs provided valuable health information and resources to their communities, improving residents' access to health services, and built trust within the community, as residents were more comfortable discussing health issues with fellow residents trained as RHAs.	

Author (year)	Countr y	Study design	Population	Type of housing	Aim	Intervention	Outcome	Results	Digital element
						and community organizing, and they became acquainted with local health sources and key health promotion organizations.<br><br>In phase 3, those successfully completed the training were hired to complete internships in their respective housing developments. They were paid to work up to six hours pr. week, and their work could include developing workshops in their respective developments or distribution and collection of health surveys.<br><br>In phase 4, the RHA program,			

Author (year)	Country	Study design	Population	Type of housing	Aim	Intervention	Outcome	Results	Digital element
						including the training program, the community impact, and former RHAs employment status, were each year evaluated.			
Grier et al. (2015)	USA	Mix-method feasibility study	Youth aged 5-17, and both youth and parent had to reside full-time at the housing authority.	Public housing.	To explore the feasibility of an experimental theory-based community garden (CG) and nutrition education program aiming at increasing fruit and vegetable consumption and gardening knowledge among youth living in public housing.	A ten-week theory-based experimental education program carried out at two youth centers of two housing authorities. Each site had access to a garden. The program was delivered by the researchers once weekly with one-hour of interactive gardening or nutrition education, followed by 30 min. of hands-on gardening.<br><br>Two reward systems were	Demand, acceptability, implementation, and limited-effectiveness testing.	Youth expressed positive impressions, especially enjoying food sampling, games, and gardening. Suggestions for improving engagement included more printed materials and door-to-door solicitation.<br><br>Parents' pre-program expectations and beliefs about gardening were generally positive, with high interest in gardening (average score of 7.85/10). Post-	No

Author (year)	Country	Study design	Population	Type of housing	Aim	Intervention	Outcome	Results	Digital element
						created, one for weekly goals, and one for program participation, to increase participants engagement. The program was offered during the operating hours for the youth center at each site. Site leaders were provided with and train in measure and record produced harvest from the garden, and basis gardening maintenance techniques to be used together with the youth those days, where the researchers were not present.		program, 87% of parents found the program timing convenient, and 53% noticed new asking behaviors in their children regarding fruits and vegetables. Most parents (93%) observed increased confidence in their children's gardening skills and would allow them to participate again.<br><br>Site leaders noted improved cohesion and positive interactions among youth, increased willingness to try fruits and vegetables (F&V), and expressed a desire to continue the program in the future.	

Author (year)	Country	Study design	Population	Type of housing	Aim	Intervention	Outcome	Results	Digital element
								Lessons were delivered as intended. The main barrier was noise and distractions from children. The key facilitator was the involvement of site leaders, whose authority helped manage the classroom effectively.<br><br>Challenges included parents answering questions for children and expressing negative opinions about certain F&V and food sampling, which potentially hindered children's willingness to try. Site leaders played a crucial role in modeling positive	

Author (year)	Country	Study design	Population	Type of housing	Aim	Intervention	Outcome	Results	Digital element
								behavior during food sampling activities.<br><br>Youth attendance averaged 4.80 and 4.40 out of 10 sessions. Out of 43 enrolled youth, 32 (74.4%) completed follow-up assessments. Significant improvements were observed in self-efficacy for asking for F&V and overall gardening knowledge. However, knowledge of food safety decreased significantly. There were no significant effects on willingness to try F&V, self-efficacy for eating or gardening F&V, other knowledge sub-categories, or overall nutrition	

Author (year)	Country	Study design	Population	Type of housing	Aim	Intervention	Outcome	Results	Digital element
								knowledge, although most non-significant effects trended positively.	
Bowen et al. (2018)	USA	Cluster randomized trial	Women in family public housing.	Public housing.	To test an environmental level diet and physical activity intervention targeting obesity among urban public housing developments.	The included developments were randomized into an intervention and control group. The intervention developments had access to all intervention activities. The control developments did not receive any intervention components.<br><br>The intervention period was one year. During that year the residents of the intervention developments could participate in any of the intervention activities;	Nutrition behavior, physical activity, self-efficacy to eat more healthfully, and BMI.	A significant change in BMI were found between the two groups. The residents in the intervention group decreased their BMI with 1.5 points on average, while the control group increased their BMIs with 0.2 points on average.<br><br>The intervention led to significant improvements in four out of five measured behaviors among the residents in the intervention group. These included increased mean fruit and	No

Author (year)	Country	Study design	Population	Type of housing	Aim	Intervention	Outcome	Results	Digital element
						Lay Health Advisors: Healthy Living Advocates (HLA) completed a 14-week training in community health outreach and a 3-day study-specific training on research processes, weight management, and patient privacy. They passed a post-training assessment before participating in the trial.<br><br>Health Screenings: Monthly screenings for blood pressure, smoking, and diabetes risk were offered. Residents received referrals to programs or healthcare providers. Screenings were		vegetable intake, a higher percentage of individuals eating fast food less than once per week, a reduction in the percentage of inactive participants, and an increase in the minutes of walking in the neighborhood per day.<br>Very minimal change were measured in the control group.<br><br>The residents from the intervention group considered the activities as useful and achievable, and the existence of the activity and the social contact in the activity were seen as helpful.	

Author (year)	Countr y	Study design	Population	Type of housing	Aim	Intervention	Outcome	Results	Digital element
						advertised for two weeks and lasted 3-4 hours, with HLAs assisting residents.<br><br>Access to Healthy Food: A van (Fresh Truck) sold fruits and vegetables weekly at each housing development. HLAs promoted and attended these sessions.<br><br>Walking Groups: HLAs led weekly walking groups, promoting them through flyers and discussions to address barriers to physical activity.<br><br>Cooking Demonstrations: Held every three months, these demonstrations provided			

Author (year)	Country	Study design	Population	Type of housing	Aim	Intervention	Outcome	Results	Digital element
						nutrition education and promoted healthy eating practices. Recipes were culturally and economically tailored, and HLAs promoted these events.<br><br>Resource Maps: Printed maps of local health-related resources were distributed by HLAs at various intervention activities.			
Freeman et al. (2020)	USA	Pre-post test	Adult residents in subsidized housing, with additional emphasis on engaging "frequent users" of healthcare services, defined as those with	Subsidized housing.	To evaluate the impact of the Health + Housing Project, a community health worker (CHW) intervention, on addressing broadly defined health	The program hired bilingual CHWs from the same neighborhood as the intervention buildings, and all shared linguistic and cultural characteristics with the residents. CHWs underwent	Assessed social and economic risk factors, health service use, and health status.	The percentage of participants reporting food insecurity decreased from 53.5% to 41.8% (P = 0.004). Additionally, the percentage of participants unable to pay rent on time decreased	No

Author (year)	Country	Study design	Population	Type of housing	Aim	Intervention	Outcome	Results	Digital element
			three or more emergency department visits or one or more hospitalizations in the past year.		needs, including social and economic risk factors.	a 35-hour training covering core competencies, chronic disease management, motivational interviewing, mental health first aid, and smoking cessation. Following this, CHWs recruited residents from all apartments, prioritizing those with frequent healthcare usage. Each CHW was initially assigned 90-100 individuals. Recruitment continued until all 450 apartments were covered. Once residents agreed to participate, CHWs conducted baseline surveys, intake assessments, goal-setting exercises,		from 22.2% to 13.2% (P = 0.009). There were also significant reductions in the percentage of participants who needed but could not access health care services, a place to exercise, job training or employment programs, and education services. In terms of health care access, more participants reported having a personal doctor post-intervention, increasing from 84.0% to 92.3% (P = 0.008). However, fewer participants reported seeing their personal doctor in the past six months, decreasing from 91.9% to 83.7% (P = 0.041). There	

Author (year)	Countr y	Study design	Population	Type of housing	Aim	Intervention	Outcome	Results	Digital element
						and developed action plans. Residents selected up to five goals from a pre-established list, rated their motivation, and worked with CHWs to create action plans. CHWs met with residents as needed, using motivational interviewing to help achieve goals. They connected residents to case management, assisted with care coordination, and provided health education on chronic disease management, nutrition, physical activity, smoking cessation, and stress reduction. Furthermore, the CHWs helped		was an increase in the number of participants reporting four or more outpatient visits, from 24.7% to 34.1% (P = 0.037). However, there was no significant change in the number of emergency department visits or hospitalizations over the past year. No significant changes were observed in self-reported general or mental health status or health behaviors.	

Author (year)	Country	Study design	Population	Type of housing	Aim	Intervention	Outcome	Results	Digital element
						residents with other needs, such as gathering documents for Supplemental Nutrition Assistance Program applications, making referrals to food pantries and Meals on Wheels, assisting with emergency rental assistance applications, and facilitating outpatient medical care by scheduling appointments and arranging transportation.<br><br>The intervention period were 15 months.			
Jassal et al. (2020)	USA	Non-randomized pilot study	Adult residents.	Public housing.	To assess the feasibility of a multi-component smoking cessation	Two resident leaders of the housing developments were recruited and underwent,	Primary outcome was weekly exhaled carbon monoxide. Furthermore, they evaluated the	No significant difference in exhaled carbon monoxide among participants before and after the	No

Author (year)	Country	Study design	Population	Type of housing	Aim	Intervention	Outcome	Results	Digital element
					program targeting smokers in public housing.	together with the research staff, a two-week tobacco cessation training program. Following this, recruitment of residents who smoke were performed by the resident leaders. Those included were able to participate in a 4-week resident leader-led cessation program undertaken in the local community center. The individual components of the intervention were delivered weekly. The components included weekly supply of nicotine replacement therapy, screening for psychosocial service needs and referrals to relevant	tobacco cessation training among resident leaders and research staff, acceptability of the intervention, participants fidelity to pharmacotherapy administration techniques, and the usage of psychosocial services.	intervention was found (10.08 vs. 9.08). 30.7 % achieved cessation based on exhaled carbon monoxide.<br><br>All resident leaders and research staffs demonstrate a score of 100 % knowledge post-tobacco cessation training.<br><br>In relation to the acceptability 26 participants had attended at least two weeks of which 10 participated in all sessions.<br><br>Only two participants consistently used transdermal NRT patches daily, while all participants used NRT gum or	

Author (year)	Country	Study design	Population	Type of housing	Aim	Intervention	Outcome	Results	Digital element
						programs, including job training and placement, personalized brief behavioral counseling as a complement to the pharmacotherapy.		lozenges multiple times per day, with less than 20% using the gum correctly at first follow-up. Patches were the only product with reported side effects, including jitteriness, nausea, itchiness, and difficulty maintaining placement. Adherence to NRTs did not correlate with biomarker-established cessation. The most frequently reported psychosocial needs were employment, housing security, substance usage treatment, mental health, and neighborhood crime. Over 80% of participants	

Author (year)	Country	Study design	Population	Type of housing	Aim	Intervention	Outcome	Results	Digital element
								desired more psychosocial services, though only 46% utilized them. Residents highlighted a preference for the accessibility of the cessation intervention and the empathy of the team, with suggestions for more frequent offerings and improved time and staff efficiency.	
Cotter et al. (2018)	USA	Pre-post test	Adult residents in Latino households.	Affordable housing.	To improve health behavior and prevent obesity through a culturally relevant obesity prevention program, Vivir Sano, which included stress reduction and behavioral lifestyle	The Vivir Sano program was a culturally tailored obesity prevention intervention designed for low-income Latino households. It consisted of 4 weekly sessions focused on evidence-based weight management	Fruit and vegetable consumption, perceived stress, social cohesion, perceived knowledge on healthy eating, participant satisfaction, and retention.	The per protocol group increased weekly vegetable consumption by approximately 4.2 servings (Cohen's d = 0.48). There was a meaningful change in knowledge about healthy eating. Over 96% of participants reported that they were likely or very	No

Author (year)	Country	Study design	Population	Type of housing	Aim	Intervention	Outcome	Results	Digital element
					intervention components, tailored to the needs of low-income Latino households.	strategies and stress reduction. The program included nutrition education based on MyPlate guidelines, identification of low-cost physical activity options, and strategies for eating healthfully on a budget. Stress management components included mindful eating, psychoeducation on the relationship between stress and health, and cognitive behavioral strategies. Sessions were conducted in English and Spanish, using culturally relevant recipes. The program also		likely to use the skills learned in the program. Approximately 85% reported that the program moderately or significantly improved their health. Out of 41 participants enrolled, 23 completed the program per protocol. The average number of sessions attended by the per protocol group was 4.09.	

Author (year)	Country	Study design	Population	Type of housing	Aim	Intervention	Outcome	Results	Digital element
						featured hands-on activities, such as preparing healthy recipes adapted from the Cooking Matters curriculum.			
Krieger et al. (2009)	USA	Pre-post test	Residents in the public housing.	Public housing.	To increase physical activities in a public housing community through the project High Point Walking and its activities.	In the development process a community assessment was performed to describe the community conditions related to physical activities.<br><br>Based on the community assessment the following interventions were implemented;<br><br>Walking groups: A 1-mile walking path around a central pond was identified as a walking trail. Five	Measures of minutes walked per day, physical activity, general health, and social connectedness.	Findings from the community assessment revealed that fewer than half of the respondents walked at least 30 min per day, and only 20% reported moderate weekly activities at recommended level. Furthermore, residents required motivation and a structured initiative to walk more frequently. Many were unaware of the health benefits of walking and the opportunities provided by the	No

Author (year)	Country	Study design	Population	Type of housing	Aim	Intervention	Outcome	Results	Digital element
						residents and six staffs from community-based organization partners were trained as walking leaders. They recruited residents older than 14 years old through flyers and word of mouth. Reminder calls, registration of participants, stretching exercises and timing of the walks were performed by the leaders. The group meet five time a week on different weekdays. Distance depended on the walker, but general the participants walked for an hour. Furthermore, the		physical environment.<br><br>The participants in the walking group significantly increased their walking from 64.6 minutes walked per day to 108.8 minutes walked per day (p-value = 0.001).<br>There were no significant changes in walking to work, school, or bus stops. Participants in the walking group that meet the recommendation of moderate weekly activity increased from 61.5% to 80.8% (p-value = 0.018).<br>Overall health showed improvement, as participants noted a reduction in the	

Author (year)	Country	Study design	Population	Type of housing	Aim	Intervention	Outcome	Results	Digital element
						participants received t-shirts, pedometers and prizes for achieving individual walking goals.<br><br>Marketing walking: A youth action team implemented a walking information campaign. A central kiosk for information sharing about health and walking was build. Additionally, a walking map of the neighborhood was composed.		number of days when their physical and mental health were suboptimal. Additionally, social connectedness enhanced significantly, evidenced by a notable rise in the average number of neighbors participants felt familiar enough with to greet while walking.	
Møller & Merrild (2020)	Denmark	Qualitative study	Residents aged 45 - 70 years living in socially, disadvantages housing, and had a high-risk health profiles.	Social housing.	To investigate the significance of participating in a preventive health check and how participation	Health checks were held at the local health center with the goal of detecting early signs or risks of disease. Participants	Topics such as the health check, health status, use of health services, daily life (including family, work, and social activities), disease	Two distinct groups emerged from the study: those who had regular contact with their GPs and those who did not. The first	No

Author (year)	Country	Study design	Population	Type of housing	Aim	Intervention	Outcome	Results	Digital element
					configured into everyday life.	identified as high-risk were advised to consult their general practitioner (GP) for further evaluation and were offered support from municipal health promotion services. The health check included both a counseling session and a physical examination, measuring blood pressure, HbA1c, cholesterol levels, weight, height, waist circumference, fitness level, and maximal oxygen consumption. Participants also completed a lifestyle questionnaire addressing diet, smoking, alcohol consumption,	history, and perspectives on health and illness were investigated. It also explored participants' needs and expectations for health care services and their interactions with their GP.	group viewed the health checks as redundant but harmless, participating out of a sense of routine or obligation. In contrast, the othergroup saw the health checks as a valuable opportunity to gain insights into their health but were ultimately disappointed by the lack of substantial support and follow-up. Participants expressed frustration with the superficial nature of the health checks, which failed to provide new information or meaningful assistance. Many were already	

Author (year)	Countr y	Study design	Population	Type of housing	Aim	Intervention	Outcome	Results	Digital element
						physical activity, and self-rated health and well-being. The results from the questionnaire and physical tests formed the basis for the health discussion. At the end of the check, participants received a written report summarizing their results.		aware of their health problems and felt that the checks did not offer the concrete help they needed. This unmet need for support was particularly pronounced among those who did not have regular GP contact. Participants struggled to implement lifestyle changes despite understanding what was necessary. Social, economic, and psychological challenges often overshadowed their ability to make these changes, underscoring the need for health interventions that consider the	

Author (year)	Country	Study design	Population	Type of housing	Aim	Intervention	Outcome	Results	Digital element
								broader context of individuals' lives.	
Reisine et al. (2021)	USA	Randomized cluster cross-over design	Residents aged ≥18, who are permanent residents of low-income senior housings, and with at least two natural teeth present.	Senior housing.	To improve oral health-related quality of life (OHRQOL) among adults through comparison of an individual-based Adapted Motivational Interviewing (AMI) intervention and a community-based campaign intervention. Furthermore, if the sequence of the interventions has influence on the effectiveness of OHRQOL, and whether clinical, demographic and	The intervention comprised two main components. The first component was Adapted Motivational Interviewing (AMI), which involved tailored counseling sessions. These sessions were based on responses to cognitive and behavioral variables identified through a survey. Participants received personalized feedback and practical training in oral hygiene techniques. Additionally, participants practiced brushing and	OHRQOL, measured by the General Oral Health Assessment Index (GOHAI). Oral Hygiene assessed using the Gingival Index (GI) and Plaque Scores (PS).<br><br>Number of missing teeth and self-reported Xerostomia.<br><br>Cognitive and behavioral factors was measured in relation to oral hygiene (e.g. oral health self-efficacy, fear of oral diseases, sugar intake)	Increased GOHAI from a mean of 39.7 at baseline to 42.3 at the end of the interventions. Both interventions were effective in improving OHRQOL, with no significant difference between the two interventions. Improvements in GI and PS were also observed, particularly following the AMI intervention. Factors such as ethnicity, health status, worries, self-efficacy, number of missing teeth, and symptoms of dry mouth were related to changes in GOHAI.	No

Author (year)	Country	Study design	Population	Type of housing	Aim	Intervention	Outcome	Results	Digital element
					psychosocial/behavioral variables affect changes in OHRQOL.	flossing skills on a typodont, a clinical model of teeth and gums, and received feedback on their performance. The second component was community-based campaigns. These campaigns were planned and executed by a Campaign Committee consisting of 6-10 resident volunteers, with support from the research staff. The campaigns included three health fairs, conducted about one month apart, featuring motivational stations, oral hygiene instruction, and presentations by oral health			

Author (year)	Country	Study design	Population	Type of housing	Aim	Intervention	Outcome	Results	Digital element
						professionals. These health fairs were open to all building residents.			
Kuross & Folta (2010)	USA	Feasibility study	Children and their parents and caregiver living at urban, minority, low-income housing.	Urban, minority, low-income housing developments.	To engage low-income, minority families in learning about healthful lifestyles through a program called the GoKids Project.	Staff spent the initial weeks meeting with tenant task forces at each housing development to understand the residents' ethnic backgrounds, nutrition and fitness needs, and preferences. The program was advertised through posters in community centers and neighborhood boards. The intervention included a participatory theater show about making healthy choices and 3 to 6 2-hour activities at the community	Rank the level of fun. Answer questions about whether they have learnt something new, and whether activities were useful at home.	The program successfully engaged families in health and fitness activities. 63% of the participants reported learning new useful information. 74% reported that they could apply the new information at home, such as increasing physical activity and incorporating more fruits and vegetables into their diets. 90% of the children ranked the activities as fun.	No

Author (year)	Country	Study design	Population	Type of housing	Aim	Intervention	Outcome	Results	Digital element
						center. Activities were designed based on input from participants and included arts and crafts, cooking, games, world traditions, science experiments, music and dance, and sports. Weekly activities were hands-on and developmentally appropriate, aimed at children from birth to 11 years old and their parents/caregivers. Teen ambassadors, who were apprentices at the museum, helped lead the activities, learning teaching and leadership skills in the process. At the end of the program, families			

Author (year)	Country	Study design	Population	Type of housing	Aim	Intervention	Outcome	Results	Digital element
						were given a free visit to the Boston Children's Museum, including free transportation. Participants received information about affordable admission options to encourage future visits.<br><br>The intervention lasted 10 weeks.			
Reisine et al. (2016)	USA	Pre-post test	Older adults and people with disabilities aged ≥62.	Senior housing.	To improve oral hygiene beliefs, attitudes and behavioral skills among older adults and adults with disabilities living in low income senior housing.	Participants underwent tailored Adapted Motivational Interviewing (AMI) sessions where they analyzed their own situations and developed plans to improve their oral hygiene. These sessions also included practical training in correct tooth brushing and	Gingival index and plaque score was measured for objective data on the oral hygiene, and 12 domains, including activities of daily living, access to oral health information, oral hygiene status, dental knowledge, hygiene behaviors, importance of oral hygiene, self-	The participants had significant improvements in oral hygiene. The mean baseline PS improved from 83% (SD 16%) to 58% (SD 31%), and the mean baseline GI improved from 1.15 (SD 0.61) to 0.49 (SD 0.46).<br><br>There was no significant change within the 12	No

Author (year)	Country	Study design	Population	Type of housing	Aim	Intervention	Outcome	Results	Digital element
						flossing techniques. Furthermore, two health fairs were organized, supported by a resident campaign committee. These fairs included oral health information booths, question-and-answer sessions with dental providers, practical oral hygiene exercises, poster contests, and health games. Additionally, a volunteer committee of residents was recruited to plan and organize campaign activities. They developed core messages about oral hygiene, which were delivered during the health fairs to	efficacy/locus of control, diet, intentions, self-management worries/fears, perceived risk, and dry mouth were assessed to report oral hygiene practices and cognitive domains.	domains, despite in the self-management fears scale from 2.2 to 1.8 ($p<0.05$), and skills in brushing and flossing from 1.9 (fair/poor) to 2.9 (good/excellent) and from 1.7 to 2.7 respectively (both $p<0.001$).	

Author (year)	Country	Study design	Population	Type of housing	Aim	Intervention	Outcome	Results	Digital element
						reinforce the AMI sessions.<br><br>The intervention took place over a period of six months.			
Marinescu et al. (2013)	USA	Qualitative study	Somali women resident in public housing.	Public housing.	To increase physical activity among women from diverse cultures where mixed-gender exercise settings are not permissible (e.g., Muslim women), through women-only programs.	A needs assessment was conducted to explore community-specific needs regardring physical activity with the aim of designing interventions that were culturally appropriate and community driven.<br><br>The "Be Active Together" (BAT) program offered free women-only exercise activities within each public housing community, and women-only	A process evaluation with the participants after the course of 18 months offering the activities.	The needs assessment revealed that it was not appropriate for the Somali women to exercise in the presence of men, and it was very important to have easy access to women-only physical activities, where they felt safe. Furthermore, they would like to keep meeting and getting to know each other.<br><br>The process evaluation revealed that the participants appreciated the opportunities of	No

Author (year)	Country	Study design	Population	Type of housing	Aim	Intervention	Outcome	Results	Digital element
						swimming opportunities through rental of a local swimming pool.<br><br>BAT staff and community resident partners advertised the classes at various community events. They used bilingual flyers, door-to-door outreach, and phone calls to boost attendance.		having women-only activities, where they felt comfortable and safe while exercising. Successful implementation due to the large number of participators was largely due to culturally mindful instructors who built trust and understood participants' cultural norms. They adapted classes to fit cultural practices, movement abilities, and available time. For Somali Muslim women, the classes provided personal time and social opportunities. Flexibility was needed for prayer times, and music	

Author (year)	Country	Study design	Population	Type of housing	Aim	Intervention	Outcome	Results	Digital element
								preferences varied among groups. Scheduling avoided cultural holidays and events was important, and childcare solutions, such as shared care, were essential when formal childcare was not available.	
Gedin & Resnick (2014)	USA	Qualitative study	Residents aged ≥55 living in low-income senior housing.	Low-income senior housing.	To increase risk awareness and promote safe sexual activity among older adults living in senior housing through a group-based educational program aimed at enhancing participants' confidence in their knowledge of sexual health	The program was called “Sexual health for older people” (SHOP). I consisted of educational workshops on sexual health, which were facilitated by an experienced public health nurse. They were held in a multiroom at each of the sites and lasted about 30 to 45 minutes. The intervention	Feedback about and experience with the workshops from the participants.	Participants found the games to be a fun, enjoyable, and effective way to reinforce learning. The structured and organized format of the sessions was appreciated, helping participants stay engaged and learn effectively. Demonstrating learned skills boosted participants' confidence and	No

Author (year)	Country	Study design	Population	Type of housing	Aim	Intervention	Outcome	Results	Digital element
					and safe sexual practices.	employed various teaching methods to accommodate different learning styles, guided by self-efficacy theory. It included verbal encouragement to promote safe sexual behavior and lectures on the basics of sexually transmitted infections and HIV transmission, supplemented with written materials. The lectures were interactive, encouraging participants to share their knowledge and beliefs, followed by accurate, up-to-date information. Topics covered included the prevalence of STIs		ability to educate others, with games helping to clarify and reinforce knowledge. The timing of the sessions was crucial, with preferences varying between sites. Some participants suggested combining the sessions with other activities or scheduling them later in the day to increase attendance.	

Author (year)	Country	Study design	Population	Type of housing	Aim	Intervention	Outcome	Results	Digital element
						and HIV among older adults, transmission modes, high-risk behaviors, symptoms, the importance of testing, and discussing sexual health with healthcare providers. Practical skills, such as applying a male condom, were demonstrated and practiced through simulations. Role-playing scenarios were used to address barriers to condom use, and a myth versus truth game reinforced the knowledge gained.			
Ahluwalia et al. (2007)	USA	A cluster-randomized dual intervention trail	Smokers residing in public and section 8	Public housing.	To increase fruit and vegetable (FV) consumption among	The intervention group received the fruit and vegetable (FV) intervention,	Fruit and Vegetable Intake, Body Mass Index (BMI), and process and	The participants in the intervention group had significant increase in FV	Yes

Author (year)	Country	Study design	Population	Type of housing	Aim	Intervention	Outcome	Results	Digital element
			housing developments.		smokers residing in public housing developments through the intervention Pathways to Health (PATH).	while the control group received a smoking cessation intervention during a period of 8 weeks.<br><br>The intervention in PATH included several key components. Participants received five sessions of motivational interviewing (MI) counseling focused on increasing their FV intake. These sessions were conducted both onsite at the housing developments and via telephone by trained counselors. Additionally, participants were provided with a bag of fresh fruits and vegetables, a	compliance evaluation.	compared to the smoking cessation group. At week 8 and month 6, the FV group reported consuming 1.58 ($p = 0.001$) and 0.78 ($p = 0.04$) more daily servings of FV, respectively, than the cessation group. At week 8 and month 6, the FV group reported consuming 3.61 ($p = 0.01$) and 3.93 ($p = 0.01$) more servings of FV in the past 30 days, respectively, than the cessation group. Participants who completed more MI sessions and tried more recipes exhibited significantly greater increases in FV intake at Month 6. No differences in BMI between the	

Author (year)	Country	Study design	Population	Type of housing	Aim	Intervention	Outcome	Results	Digital element
						cookbook containing healthy recipes, dietary education materials, and two videos on FV consumption. These materials were designed to enhance self-efficacy and outcome expectations, addressing barriers to FV consumption and emphasizing the health benefits of FV. The intervention was guided by Social Cognitive Theory and the Health Belief Model, which helped design the educational materials and counseling sessions to improve participants' confidence in their		groups were observed. The majority of participants reported being very satisfied with the FV intervention and believed that the program had a significant influence on their FV consumption.	

Author (year)	Countr y	Study design	Population	Type of housing	Aim	Intervention	Outcome	Results	Digital element
						ability to increase FV intake and their belief in the health benefits of doing so.<br><br>The control group received an 8-week supply of nicotine gum, instructions for using the gum, and educational materials related to quitting smoking. The control groups also received five sessions of MI counseling tailored to their smoking cessation.			
Brown et al. (2011)	USA	Mixed-method	Public housing residents.	Public housing.	The program, called Healthy Families Brooklyn (HFB), aims to decrease health disparities by increasing access to care, improving	The program focused on improving access to healthcare, providing health education, and facilitating navigation of the healthcare system through the use of	Measure HFAs understanding of health topics and evaluate the effectiveness of the training and the impact of the HFAs' work on the community.	The HFAs successfully increased their health knowledge, particularly in areas such as diabetes and asthma, as evidenced by significant	No

Author (year)	Countr y	Study design	Population	Type of housing	Aim	Intervention	Outcome	Results	Digital element
					health education, and easing navigation of the healthcare system. HFAs aimed to improve health outcomes and reduce health disparities in underserved communities.	Community Health Workers, referred to as Healthy Families Advocates (HFAs). HFAs are lay health workers from the community who were trained to help residents access healthcare and social services. HFAs underwent a comprehensive training program that includes 30 hours of classroom instruction. Training covered topics such as community health empowerment, health access and outreach, public health, disease-specific knowledge (e.g., asthma, diabetes, hypertension), and		improved scores ($p < 0.01$) from a mean of 17.70 to a mean of 19.90. The HFAs experienced to effectively built trust and foster relationships with residents, which facilitated better health outcomes. HFAs reported enhanced access to healthcare services, improved health behaviors, and greater health knowledge among the residents.	

Author (year)	Countr y	Study design	Population	Type of housing	Aim	Intervention	Outcome	Results	Digital element
						communication skills. The HFAs conducted health presentations and outreach activities within the community. They provided residents with information on prevalent health issues, assisted them in accessing public benefits, and helped navigate healthcare services. HFAs also offered personalized support by meeting residents in their homes, helping to schedule medical appointments, access free or reduced-cost prescription drugs, and providing educational materials on			

Author (year)	Country	Study design	Population	Type of housing	Aim	Intervention	Outcome	Results	Digital element
						various health topics.			
Aselton (2011)	USA	Qualitative study	Elderly and disabled people living in public senior housing.	Public senior housing.	To increase the quality of life of elderly and disabled residents in public housing and improve their access to wellness activities.	Each semester (twice a year), the Wellness Program received six to eight nursing students. They conducted a needs assessment to determine the residents' health education and service needs, and to determine which activities would be relevant to include in the Wellness Program. The program offered regular health screenings, such as blood pressure monitoring, and provided health education on topics like secondhand smoke, depression, diabetes, men's health, herbal	The residents' perceptions of the Wellness Program and a biannually review of the experience of the nursing students through poster sessions.	Residents reported high satisfaction with the various activities, including health screenings, exercise programs, and home visits. They benefited from increased access to health education, social support, and personalized health assessments, which contributed to improved health and well-being. Nursing students gained valuable community health experience, enhancing their skills in health education and promotion.	No

Author (year)	Country	Study design	Population	Type of housing	Aim	Intervention	Outcome	Results	Digital element
						medicine, falls prevention, food safety, and nutrition. Exercise programs were also a key component, featuring gentle exercises, relaxation therapy, meditation sessions, tai chi, music therapy, and seated jazz dancing to accommodate residents with varying levels of mobility. The activities were held approximately once a week. In addition to these activities, the program included home visits for residents unable to attend community room events. During these visits,			

Author (year)	Countr y	Study design	Population	Type of housing	Aim	Intervention	Outcome	Results	Digital element
						nursing students conducted health assessments, medication reviews, and provided social support. The program also featured a special Spa Day at the end of each semester, where residents could enjoy foot soaks, hand massages, facials, and nail care.<br><br>The program had run in five years.			
Lai et al. (2017)	China	Mixed methods	Residents in a public low rent housing.	Public Housing.	To enhance the residents' family well-being, and neighborhood cohesion through a series of community activities.	A train-the-trainer (TTT) workshop was implemented for resident leaders to become health promotors in the Learning Families Project. Following, the health promotors implemented different health promoting	The residents perceived personal mental and physical health, family communication behaviors, family well being, and neighborhood cohesion.	At one year, the residents had a significant increase in physical and mental health, and the practice of positive communication behaviors and better neighbor cohesions increased as well.	No

Author (year)	Country	Study design	Population	Type of housing	Aim	Intervention	Outcome	Results	Digital element
						activities in their public housing area to engage residents to join activities and learn with their family members, and support after a training session.			
Agarwal et al. (2019)	Canada	Cluster-randomized controlled trial	Low-income older adults (≥55 years) living in public housing.	Public housing.	The main objective was to assess the variation in average Emergency medical service (EMS) calls at the building level, comparing intervention and control buildings across various community locations. Additionally, the secondary objective included evaluating individual-level	Community Paramedicine clinic is a weekly drop-in program offering one-to-one assessments for blood pressure, diabetes, and fall risk, along with health education, promotion, and referrals to community resources (e.g., dietitian-led cooking classes, smoking cessation programs). Community paramedics, trained through online modules	The primary outcome at the building level was the number of EMS calls per 100 apartment units per month. Secondary outcomes at the individual level included changes in blood pressure (BP), lifestyle risk factors (such as physical activity, fruit and vegetable intake, and body mass index [BMI]), health-related quality of life (HRQoL), and quality-adjusted	No significant difference in EMS calls between intervention and control buildings. However, sensitivity analysis excluding data from two building pairs with eligibility changes revealed a significant reduction in EMS calls in the intervention buildings, with a mean difference of 0.90 calls per 100 apartment units per month (95% CI: 1.54 to 0.26).	No

Author (year)	Country	Study design	Population	Type of housing	Aim	Intervention	Outcome	Results	Digital element
					enhancements in health-related quality of life (HRQoL) and chronic disease risk factors among older adults residing in the intervention buildings, in contrast to those living in the control buildings.	and webinars, conducted these sessions in common areas of the buildings. They used the Canadian Diabetes Risk Questionnaire (CANRISK) to assess participants, repeating it every six months, and provided further testing and referrals based on risk levels. High-risk participants were directed to appropriate health care services, while those at moderate risk were referred to community services to manage chronic disease risk factors.<br><br>The usual care control group	life years (QALYs).	Residents in the intervention group experienced significant health improvements, including a decrease in both systolic (3.65 mmHg) and diastolic (2.03 mmHg) blood pressure. Additionally, there were positive changes in lifestyle risk factors, such as increased physical activity, better fruit and vegetable consumption, and a reduction in body mass index (BMI). Health-related quality of life (HRQoL) also improved, with a notable increase in quality-adjusted life years (QALYs) by 0.06.	

Author (year)	Country	Study design	Population	Type of housing	Aim	Intervention	Outcome	Results	Digital element
						included services that residents could access by visiting their family physician, as well as ongoing services provided within their building by local community agencies.			
Allen et al. (2020)	USA	Feasibility study	Women aged 18-26 years old, who were living in public housing.	Public housing.	To access the feasibility and initial impact of a month-long Twitter campaign promoting knowledge about the HPV vaccination targeting a group of low-income and racially/ethnically diverse women.	Over a period of one month, women received daily tweets featuring educational content developed by the Centers for Disease Control and Prevention, the National Cancer Institute, and the Massachusetts Department of Public Health. These messages primarily focused on HPV vaccination but also included information	Changes in HPV knowledge, attitudes, perceived risk for cervical cancer, perceived barriers to HPV vaccination, decision self-efficacy, and vaccination intentions. Additionally, Twitter analytics were used to track engagement metrics such as likes and retweets. Monitoring the number of participants who remained	Twitter was an acceptable method for delivering health messages, with most participants finding it a suitable educational strategy. However, there were no significant changes in HPV knowledge, perceived benefits or barriers to vaccination, decision self-efficacy, or vaccination intentions after	Yes

Author (year)	Countr y	Study design	Population	Type of housing	Aim	Intervention	Outcome	Results	Digital element
						promoting cervical cancer screening.	engaged with the campaign, did not block messages, completed the post-test survey, and the acceptability of receiving health messages via Twitter.	the campaign. The only notable change was a decrease in perceived risk for cervical cancer. Despite high engagement with the campaign, the intervention did not significantly impact the participants' health behaviors or intentions regarding HPV vaccination.	
Deville-Stoetzel et al. (2021)	Canad a	Mixed Method	Older adults (aged ≥ 60) living in social housing.	Social housing.	To improve cardiovascular health through the management and prevention of hypertension with the program Cardiovascular Health Awareness Program (CHAP),	The CHAP included monthly educational sessions with workshops on various health topics (e.g. diabetes, diet, physical activity, and hypertension), and health assessments conducted by trained volunteers. These	The study measured the relationships among residents in two buildings with different CHAP attendance rates. This included indegree centrality, total degree centrality, and group membership to identify opinion leaders and bridging	This study found that central leaders significantly influenced participation, with high attendance linked to active leaders and low attendance linked to inactive leaders and prevalent conflicts. Social network analysis identified key leaders and	Yes

Author (year)	Country	Study design	Population	Type of housing	Aim	Intervention	Outcome	Results	Digital element
					targeting older adults.	assessments included blood pressure measurements and cardiovascular risk evaluations, with results shared with participants' physicians. Recruitment strategies involved posters, flyers, and automated reminders. The program aimed to increase health awareness, promote healthy habits, and support self-management among residents.	individuals. Additionally, everyday life, relationships with neighbors, family, and friends, and reasons for attending or not attending CHAP sessions were explored.	highlighted the impact of interpersonal dynamics on participation. Residents used various strategies to manage relationships, often avoiding activities due to fear of gossip and conflicts. The study recommended strengthening confidentiality, using peer networks for recruitment, encouraging attendance in pairs, and involving positive leaders or bridging individuals to enhance participation.	
Srivarathan et al. (2020)	Denmark	Qualitative study	Residents aged ≥45, born in Denmark or Turkey, living	Social housing.	To enhance social relations among residents in a	The intervention was co-created with residents, involving a	Residents' engagement, motives, perceived	Residents were motivated to participate to establish and	No

Author (year)	Countr y	Study design	Population	Type of housing	Aim	Intervention	Outcome	Results	Digital element
			in the deprived social housing.		deprived social housing area through fostering a sense of belonging and improve social cohesion.	community seminar and coordination meetings. Activities included bus trips, with guided tours, lunch, and afternoon tea. The bus trips were social outings to different sites and historical landmarks in Denmark.	outcomes, and barriers. Furthermore, interactions and dynamics during the intervention were documented.	enhance social relations and to explore the world outside their housing area. The intervention provided a break from their routine and an opportunity to experience new places and activities. Participants reported positive social interactions, forming new relationships, and strengthening existing social ties, fostering a sense of community. However, cultural and language differences posed significant challenges, leading to suboptimal interactions between residents of different ethnic backgrounds.	

Author (year)	Country	Study design	Population	Type of housing	Aim	Intervention	Outcome	Results	Digital element
								Additionally, structural changes and territorial stigmatization, such as the planned demolition of apartments, created uncertainty and reduced engagement.	
Schwinn et al. (2014)	USA	Randomized controlled trial	Adolescent girls aged 10 - 12 and their mothers.	Public housing.	To improve dietary intake, increase physical activity, and reducing drug use risks among adolescent girls aged 10 to 12 years and their mothers through a web-based, family-involvement health promotion program.	The intervention consisted of a three-session web-based health promotion program, completed by mother-daughter dyads over a three-week period. The sessions covered topics such as active listening, communication, the benefits of family meals, drug knowledge, rule-setting, making healthy decisions, coping	Measures of mother-daughter closeness, communication, parental monitoring, substance use, fruit and vegetable intake, physical activity, perceived stress, and drug refusal skills.	Girls in the intervention group reported better communication with their mothers and increased parental monitoring compared to the control group. Mothers in the intervention group also reported better communication and closer relationships with their daughters, as well as increased vegetable intake	Yes

Author (year)	Country	Study design	Population	Type of housing	Aim	Intervention	Outcome	Results	Digital element
						skills, and problem-solving. Each session was approximately 25 minutes long.		and physical activity.<br><br>The girls in the intervention group reportee better communication and closer relationships with their mothers, reduced stress, better drug refusal skills, and increased fruit intake. Mothers continued to report increased parental monitoring.	
Oliver et al. (2024)	Australia	Mixed-method evaluation	Residents in distinct high-rise public housing.	Public housing.	To deliver place-based peer-to-peer COVID-19 education to residents of high-rise public housing to improve residents' engagement with health services and	The intervention consisted of the cohealth Health Concierge program, which operated from July 2020 to June 2022. The program involved placing peer-to-peer health educators, known as Concierges, in	Residents' satisfaction of the interaction with the Cocierges. Furthermore, assessing program reach, effectiveness, and adoption, as well as residents' trust in public health authorities and	The Health Concierge program had a significant reach and impact. 75 % of the residents reported receiving information about COVID-19 testing and vaccination from a Concierge, and nearly two-thirds had spoken	No

Author (year)	Countr y	Study design	Population	Type of housing	Aim	Intervention	Outcome	Results	Digital element
					public health activities, promote COVID-19-safe behaviors, and provide community support during the COVID-19 pandemic.	the foyers of 31 residential high-rises. The Concierges, many of whom were bicultural and multilingual, provided residents with up-to-date information about COVID-19, public health restrictions, and health services, including testing and vaccination. They also offered support and organized community wellbeing activities. Concierges received training in COVID-19 safety, de-escalation, and emotional intelligence, and participated in daily debriefing sessions with on-	COVID-19 vaccines.	with a Concierge in the previous six months. The program was particularly valued during the initial phase of the pandemic, with Concierges providing essential support and information. However, as public health restrictions eased, some residents felt the program's relevance diminished. Despite this, the median satisfaction score for the Concierge service was 10 out of 10. Engagement with Concierges varied, with some residents feeling a strong connection and others perceiving limited interaction. The	

Author (year)	Country	Study design	Population	Type of housing	Aim	Intervention	Outcome	Results	Digital element
						site nurse managers.		program faced challenges related to unclear management and hiring criteria, leading to variable service delivery. Concierges expressed a need for more comprehensive training and clearer role definitions.	
Whittemore et al. (2014)	USA	Mixed-method	Adults >21 years of age, at-risk for T2D (2 or more risk factors, such as overweight, age, family history of T2D), and live in public housing.	Public housing.	To promote healthy behaviors and prevent type 2 diabetes (T2D) among residents to improve health outcomes in a population at high risk for T2D due to socioeconomic and racial/ethnic disparities through a modified Diabetes	The mDPP included seven interactive educational sessions on nutrition and exercise aimed at preventing type 2 diabetes (T2D). These sessions also covered topics such as low-fat eating, recipe adjustments, and overcoming exercise barriers. The first two sessions were the	Residents' attendance, attrition, and adherence to the protocol. Nurses recorded each session's details, including attendance, duration, content, protocol adherence, and any deviations. Additionally, identifying barriers and facilitators to implementation.	Attendance was found to be suboptimal, with 60% attendance in the enhanced standard care group and 54% in the diabetes prevention group. Notably, attendance was higher during the first three months at 74%, but it dropped to 34% in the latter three months. The attrition rate at six months was 27%,	No

Author (year)	Country	Study design	Population	Type of housing	Aim	Intervention	Outcome	Results	Digital element
					Prevention Program (mDDD).	same as those provided to the enhanced standard care control group. Additionally, the mDPP offered behavioral support in goal setting, self-monitoring, and problem-solving to address barriers to change. The key difference between the two programs was the extra content provided by the mDPP and the behavioral support offered throughout the six-month program duration. Classes were taught in English with Spanish translation available, family		with 20% of participants lost to follow-up and 7% moving away.<br><br>In terms of protocol adherence, the overall implementation was 83% across all classes and groups. The enhanced standard care group had a slightly higher adherence rate of 84% compared to 80% in the diabetes prevention group. Protocol adherence was generally above 70% for all classes, except for one class where external disruptions occurred.	

Author (year)	Country	Study design	Population	Type of housing	Aim	Intervention	Outcome	Results	Digital element
						members were invited to attend, and childcare was provided. Two homecare nurses were hired to implement the program and provide the classes to residents. Classes were initially held every other week for the first month and then monthly. To encourage participation, classes were offered at various times, and participants received a schedule. Community health workers (CHW) reminded participants about upcoming classes and encouraged attendance. If a participant missed a class, handouts were delivered to		The study identified several barriers to implementation, including inconsistent attendance, personal issues among CHWs, and logistical challenges such as obtaining access to community centers and ensuring reliable childcare. Despite these challenges, there were also several facilitators that contributed to the program's success. These included the enthusiasm and resourcefulness of nurses and CHWs, effective training, and the use of interactive and culturally tailored educational materials. Nurses and CHWs	

Author (year)	Country	Study design	Population	Type of housing	Aim	Intervention	Outcome	Results	Digital element
						their home or mailed, and follow-up phone calls or handwritten notes were sent to encourage attendance at the next class.		reported that the interactive group education and simplified content were well-received by participants.	
Velez et al. (2023)	USA	Multi-phased mix-method design	Adult residents of two family designated public housing, who spoke either English or Spanish.	Public housing.	To reduce the intake of sugar-sweetened beverages and foods (SSBF) among adult residents of public housing through a photo-enhanced and theory-based health promotion messages.	Firstly, a needs assessment was conducted with the residents. through a modified photovoice methodology. It covered barriers and facilitators in reducing SSBFs. Secondly, 15 health promotion messages with accompanying photos including advise and web links to reduce SSBF consumption was developed. Messages were short (140 characters) and	Acceptability of the developed messages and screening of SSBF consumption.	The average ratings for each message across different domains, such as encouragement and satisfaction, ranged between 4.65 and 4.83 on a 1 to 5 scale. Messages aimed at boosting motivation received the lowest scores overall. Specifically, the scores for message relevance and encouragement were lower compared to those for satisfaction,	Yes

Author (year)	Country	Study design	Population	Type of housing	Aim	Intervention	Outcome	Results	Digital element
						delivered via print, SMS text, or social media. The messages targeted self-efficacy, motivation, and outcome expectations, and were developed iteratively with community input. The messages was delivered in a period of one month.		understanding, and appeal. Messages delivered via social media had the lowest average scores (4.71), followed by text messages (4.76), and paper-based messages (4.81). The status of caregivers influenced the scoring; caregivers rated messages involving activities with children, like preparing home-cooked meals, most favorably. In contrast, non-caregivers rated messages about using SNAP benefits to save money highest. Both groups rated messages about reducing dietary sugars the lowest.	

Author (year)	Country	Study design	Population	Type of housing	Aim	Intervention	Outcome	Results	Digital element
								Most participants indicated that the messages met all (32.4%) or some (64.9%) of their goals. Additionally, 94.6% of participants found the number of messages appropriate, and 82.9% found them useful. A majority (81.1%) indicated they would likely share the messages with others if they were made available to the community.	
